# Involvement of Human Cellular Proteins and Structures in Realization of the HIV Life Cycle: A Comprehensive Review, 2024

**DOI:** 10.3390/v16111682

**Published:** 2024-10-29

**Authors:** Alexandr N. Schemelev, Vladimir S. Davydenko, Yulia V. Ostankova, Diana E. Reingardt, Elena N. Serikova, Elena B. Zueva, Areg A. Totolian

**Affiliations:** St. Petersburg Pasteur Institute, St. Petersburg 197101, Russia; vladimir_david@mail.ru (V.S.D.); shenna1@yandex.ru (Y.V.O.); dianavalutite008@gmail.com (D.E.R.); genista.bio@gmail.com (E.N.S.); ezueva75@mail.ru (E.B.Z.); totolian@pasteurorg.ru (A.A.T.)

**Keywords:** human immunodeficiency virus, HIV, HIV life cycle, HIV proteins, cellular partners

## Abstract

Human immunodeficiency virus (HIV) continues to be a global health challenge, with over 38 million people infected by the end of 2022. HIV-1, the predominant strain, primarily targets and depletes CD4+ T cells, leading to immunodeficiency and subsequent vulnerability to opportunistic infections. Despite the progress made in antiretroviral therapy (ART), drug resistance and treatment-related toxicity necessitate novel therapeutic strategies. This review delves into the intricate interplay between HIV-1 and host cellular proteins throughout the viral life cycle, highlighting key host factors that facilitate viral entry, replication, integration, and immune evasion. A focus is placed on actual findings regarding the preintegration complex, nuclear import, and the role of cellular cofactors such as FEZ1, BICD2, and NPC components in viral transport and genome integration. Additionally, the mechanisms of immune evasion via HIV-1 proteins Nef and Vpu, and their interaction with host MHC molecules and interferon signaling pathways, are explored. By examining these host–virus interactions, this review underscores the importance of host-targeted therapies in complementing ART, with a particular emphasis on the potential of genetic research and host protein stability in developing innovative treatments for HIV/AIDS.

## 1. Introduction

More than forty years have passed since the first reports of patients with acquired immunodeficiency syndrome (AIDS) [1]. Three years after that, the cause of the disease was identified as the human immunodeficiency virus (HIV), a lentivirus that destroys the immune system. HIV-1 infects and destroys ‘cluster of differentiation 4’ (CD4^+^) lymphocytes. Unchecked, this reduces the patient’s ability to resist normally non-pathogenic or weakly pathogenic microorganisms, such as pneumocystis, candida, cytomegalovirus, and several other opportunistic infections. If untreated, these infections and some rare cancers contribute to further immune system collapse and patient death.

At the end of 2022, more than 38 million people worldwide were living with HIV [2]. Although no effective vaccine has yet been developed, extensive research on inhibiting various stages of the HIV life cycle has paved the way for the development of new antiretroviral drugs [3]. Despite advances in highly active antiretroviral therapy (HAART), treatment can lead to the development of drug toxicity and resistance [4]. HAART is also implicated in the occurrence of adverse metabolic effects such as dyslipidemia, high blood pressure, and insulin resistance [5]. These aggravating factors emphasize the need for new, less toxic, more effective, and additional complementary therapeutic approaches.

More than twenty years ago, the human genome was sequenced, and sequences also became available for many of the most enigmatic infectious diseases, both chronic and fatal. This has enabled the study of HIV using new tools of genomic analysis [6]. HIV shows considerable epidemiological heterogeneity, much of which can be attributed to host genetic factors. Beginning in the early 1980s, epidemiologists began to assemble longitudinal cohorts of populations at risk for AIDS to describe this heterogeneity. Many of them collaborated with geneticists who, using association analyses based on population genetics, identified genes with naturally occurring variants that influence HIV infection, the dynamics of AIDS progression, and HAART outcomes.

The identification of genes that influence the HIV infectious process is key to unraveling HIV–host interactions for drug and vaccine development. In 1996, a 32 base pair deletion (CCR5 Δ32) was discovered that conferred almost complete protection against HIV infection in homozygotes [7,8]. This was the first conclusive evidence that transmissible strains of HIV preferentially utilize the C-C chemokine receptor type 5 (CCR5) coreceptor for cell entry, and led to the development of a new class of anti-HIV drugs that inhibit HIV cell entry [7]. In 2007, an HIV-1-positive patient was cured after transplantation of CCR5 Δ32/Δ32 stem cells [9]. This case illustrates the potential of translational genetic research in the fight against HIV/AIDS. Unlike HIV, which can develop resistance through elusive mutations, human cellular proteins are relatively stable. Therefore, the search for host genetic factors associated with the development of HIV infection is becoming increasingly active.

This review will attempt to update the status of known interactions between viral and host proteins, taking into account reviews that have appeared to date [10,11,12,13,14]. We will highlight those host genetic influences that are plausible, replicated and involved in HIV disease, while discussing their role in disease progression and HIV infectivity.

## 2. Genetic and Antigenic Structure of HIV

**Figure 1:**
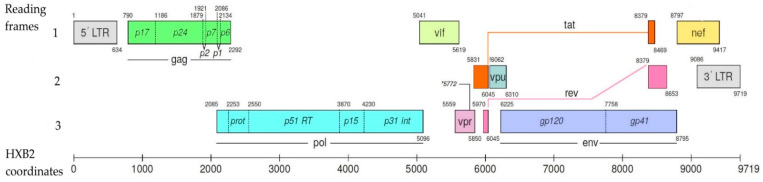
Layout of the HIV genome based on information from the HIV Sequence Compendium 2021. Numbers indicate HXB2 coordinates.

According to the current understanding detailed in the HIV Sequence Compendium 2021 [15], the HIV genome is diploid, i.e., it is represented by two molecules of single-stranded, positive-sense RNA. The HIV genome contains two genes encoding structural proteins (*gag*, *env*), a *pol* gene encoding viral enzymes, and six genes encoding regulatory proteins (*tat*, *rev*, *nef*, *vif*, *vpr*, *vpu*). In total, the genome encodes six structural proteins, three enzymes, and six regulatory proteins (Figure 1).

The *gag* gene encodes a polyprotein, the precursor of p55, which is cleaved by viral protease into the structural proteins p17, p24, p2, p7, p1, and p6. The matrix protein p17 forms the core of the viral particle. The capsid protein p24 forms the cone-shaped capsid of the virion. Proteins p1 and p2 are linker peptides. Protein p7 forms the virion nucleocapsid (the envelope covering the RNA). Core protein p6 participates in the final stages of virus formation by binding the constricted part of the capsid to the lipid envelope of the virion.

The *env* gene encodes the protein gp160, which is cleaved by the cellular endoprotease furin in the endoplasmic reticulum into the surface glycoprotein gp120 and the transmembrane glycoprotein gp41. These viral glycoproteins form a complex (through non-covalent interactions) called Env or, more generally, envelope protein. The architecture of Env consists of its spike-shaped head (10–15 nm wide) composed of gp120, a relatively short stem (~10 nm) composed of gp41, and a central void surrounding the C3 axis [16].

It is known that gp120 contains five relatively conserved domains (C1–C5) and five variable loops (V1–V5), named after their relative genetic heterogeneity. Each of the variable domains, with the exception of V5, consists of a loop structure formed by a disulfide bond at its base. Variable loops lie predominantly on the surface of gp120 and play a crucial role in evading immune response and binding to coreceptors, especially the V3 loop [17].

The *pol* gene encodes the following enzymes: reverse transcriptase and RNase H (p66/51); integrase (p32); and protease (p10). Reverse transcriptase (RT) has RNA-dependent DNA polymerase and RNase activity. The *tat* gene determines the synthesis of transcription activator (p14), which enhances RNA polymerase II-mediated elongation of integrated viral cDNA, as well as stimulates transcription of proviral DNA and RNA transport from the nucleus to the cell cytoplasm. The *rev* gene determines the synthesis of a regulator of viral gene expression (p19), which accelerates the escape of viral RNA from the nucleus into the cell cytoplasm and switches the synthesis of regulatory proteins to the synthesis of structural proteins.

The *nef* gene is responsible for the synthesis of an effector protein (p27/25) that enhances the infectious properties of virions. In particular, this protein suppresses the expression of CD4 and major histocompatibility complex (MHC) molecules on the surface of infected cells. The *vif* gene determines the synthesis of viral infectious factor (p23), which promotes viral replication. Strains lacking the *vif* gene penetrate into target cells but do not replicate in them, as viral DNA synthesis remains incomplete. The *vpr* gene determines the synthesis of viral protein R (p15). Proposed functions for Vpr include: targeting pre-integration complexes; arresting cell growth; transactivating cellular genes; and inducing cellular differentiation. The *vpu* gene encodes the synthesis of viral protein U (p16), which promotes CD4 degradation and enhances virion release from the cell.

In HIV, the main antigens are group-specific and species-specific antigens (core or nuclear antigen p24), as well as type-specific antigens (envelope antigens gp41, gp120). HIV is characterized by high antigenic variability. As a result of RT failures, serologically different viral clones can be isolated from a patient.

## 3. The Viral Life Cycle and Its Interaction with Host Proteins

**Figure 2:**
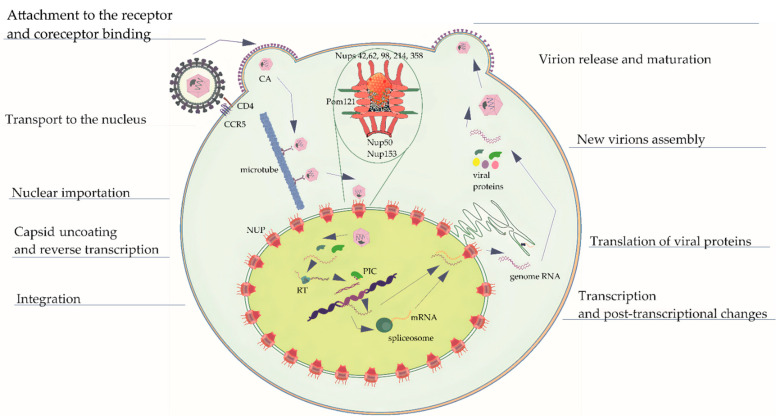
Overview of the HIV life cycle based on evidence in the literature.

The HIV life cycle (Figure 2) can be divided into 11 phases: binding/attachment; fusion; transport; nuclear import; reverse transcription; integration; transcription/translation; assembly; budding; release; and maturation. However, some additional life cycle phases can be considered that are equally important, such as mRNA splicing and post-translational changes in amino acid sequences. The life cycle stages are carried out by viral proteins in association with host proteins.

### 3.1. The Stage of Viral Attachment to the Receptor and Coreceptor Binding

Initially, the viral envelope glycoprotein, gp120, interacts with the CD4 receptor on the surface of T cells, which causes a conformational change in gp120, allowing it to bind to a coreceptor: either C-X-C chemokine receptor type 4 (CXCR4) or CCR5.

Env binding to CD4 causes V1/V2 and then V3 rearrangements. In addition, CD4 binding leads to the formation of a bridging sheet, a four-chain β-sheet consisting of two double-chain β-sheets that are spatially separated in the unliganded state. The linked sheet and the displaced V3 loop play a crucial role in the next step of viral entry, the interaction with coreceptors [18,19]. In the bound state of gp120, the V3 loop of the external domain extends to the Env surface and becomes available for interaction with coreceptors. It is currently known that HIV-1 binds to CXCR4 if the 11th and 25th positions of V3 are positively charged. Otherwise, HIV-1 uses CCR5 as a coreceptor [20,21].

Binding to the coreceptor induces the hydrophobic gp41 peptide to converge to the cell membrane, allowing it to incorporate into the host cell membrane. This binds the virus and host membranes, allowing the fusion peptide of each gp41 in the trimer to fold in a hinge region, bringing the amino-terminal helical region (HR-N) and carboxy-terminal helical region (HR-C) out of each gp41 subunit, causing them together to form a group of six helices (6HB). Since the HR-N domain is in close proximity to the host cell membrane due to the fusion peptide, and the HR-C domain is in close proximity to the viral membrane due to the transmembrane domain of gp41, the driving force is the formation of 6HB. This brings the opposing membranes into close proximity, resulting in the formation of a fusion pore. Thus, binding to the coreceptor unlocks the potential energy of the gp41 fusion complex, leading to the formation of 6HB, opening and stabilization of the membrane fusion pore, and subsequent delivery of the viral content into the host cell cytoplasm [22,23,24].

To date, it is also known that gp120 can interact with integrin proteins in intestinal lymphoid tissue. An epitope in the V2 loop mediates the interaction between gp120 and α4β7. The signal transduction produced by the gp120–α4β7 interaction leads to the activation of LFA-1, an event that is crucial for the formation of immunological synapses. This cascade of interactions promotes infection in intestinal lymphoid tissue during the early stages of infection, with subsequent depletion [25,26].

### 3.2. Transport of Nucleocapsid to the Nucleus

HIV-1 requires microtubule (MT)-based transport and dynein to reach the nucleus, but it does not bind directly to MT motor proteins [27,28]. Instead, fasciculation and elongation protein zeta 1 (FEZ1), a multifunctional adaptor of kinesin-1, binds to incoming HIV-1 capsids, allowing the virus to regulate its retrograde mobility [29]. Mechanistically, negatively charged polyglutamate sites in FEZ1 bind to a ring of positively charged arginine residues in the central pore of the capsid hexamer, facilitating the movement of incoming viral core. HIV-1 also utilizes microtubule affinity regulating kinase 2 (MARK2) [30] to phosphorylate FEZ1 by serine 58 (S58), which plays a crucial role in controlling kinesin-1-based transport to the nucleus [29,31]. Although kinesin-1 normally mediates outward movement of cargo, capsid-associated FEZ1 allows HIV-1 to control the balance of forward and backward (bidirectional) movement of viral particles and their eventual net movement forward to the nucleus for efficient infection.

Shortly after the discovery of FEZ1, it was discovered that bicaudal D cargo adaptor 2 (BICD2) acts as an HIV-1 capsid-specific adaptor for dynein-mediated motility of incoming viral core [32,33]. Consistent with their roles as respective adaptors for kinesin-1 and dynein, both FEZ1 and BICD2 are required for retrograde transport and disassembly of incoming capsids during early HIV-1 infection [29,31,32,33].

### 3.3. Stage of Nuclear Import

To fully realize the viral life cycle, the intact nucleocapsid docks with the nuclear pore complex (NPC) and passes through it into the nucleus with an intact or nearly intact capsid. The NPC is the largest macromolecular complex in the human cell (~110 MDa), consisting of ~30 different nucleoporins (NUPs). Each NUP has 8–48 copies in the NPC, which together form a basket-shaped structure [34,35,36,37] that contains a selective barrier in the central channel [38,39,40]. The selectivity barrier at the entrance to the core of the central channel is formed from intrinsically disordered NUP domains that are enriched in phenylalanine-glycine motives (FGM). These FGM domains are found in about one-third of NUPs.

The central channel diameter of the human NPC [41] was initially thought to be ~40 nm, allowing for the transport of cellular cargo [38,39,40], but was thought to be too narrow for the passage of an intact HIV-1 core. However, when analyzing cryo-electron tomograms of SupT1-R5 cells, irrespective of infection status, some NPCs were seen to feature a significantly larger diameter of ~64 nm, which exceeds the width of the HIV-1 capsid (~55–60 nm) [42]. Whether the ~64 nm width of the central channel is universal, or reflects its natural state in other eukaryotic cell types, remains unknown. However, a report showed that intact HIV-1 capsid was found in the nucleus of a wide range of cells other than SupT1-R5 [43].

It is currently unclear how the HIV capsid can overcome the NPC selectivity barrier if its size is more than 1000 times the passive diffusion limit. There is a model in which the HIV capsid mimics the karyopherin mechanism of NPC transit by dissolving in the diffusion barrier through specific, multivalent FGM interactions [44].

Early capsid uncoating can occur within the first hour of infection [45,46,47], whereas only an intact capsid delivers its genome to the nucleus and completes integration for successful infection [42,46,47]. Although a focus on intact viral particles that lead to successful infection is warranted, some early or intermediate states of particles imported in a partially degraded state may still be biologically relevant.

### 3.4. Reverse Transcription

Reverse transcription appears to occur after nuclear import, but to date it is not known whether it is associated with the release of capsid contents. It is known that the nucleocapsid reaches nuclear speckles at the completion of reverse transcription; it approaches the site of integration and releases complementary DNA (cDNA) [48,49,50,51]. Reverse transcriptase is an asymmetric heterodimeric enzyme consisting of a 560 amino acid 66 kDa subunit (p66) and a 440 amino acid 51 kDa subunit (p51). The heterodimer contains one active DNA polymerization site, an RNase H active site, and an RNA binding site.

However, it has been shown quite reliably that RT acts in complex with CA, forming together with it and other structures a reverse transcriptase initiation complex (RTIC). This complex of proteins and nucleic acids is required for the first step of reverse transcription, namely primer initiation and annealing. Since the HIV structure lacks the primer necessary for the initiation of reverse transcription, the complex contains recruited human tRNA_3_^Lys^, which is stored inside the viral particles [52]. The cellular tRNA primer is packaged into particles by interacting with Gag with lysyl synthetase (LysRS), which has bound tRNA_3_^Lys^ [53]. Additional specificity is achieved because only those LysRS molecules that have bound tRNA_3_^Lys^ are recruited by additional interactions with the thumb and Pol connecting domains (in the Gag-Pol precursor) [54]. To initiate reverse transcription, the 3′-terminal 18 nucleotides of primer tRNA are annealed to a complementary viral sequence, called a primer binding site (PBS), present in the 5′-untranslated region (UTR) of genomic RNA. An important role in RTIC binding is played by p7, which functions as a nucleic acid chaperone and also binds CA to viral RNA (vRNA), as well as taking part in annealing and formation of the vRNA/tRNA_3_^Lys^ complex.

The interaction between vRNA and tRNA forms the RNA architecture that is required for the initiation of reverse transcription. This complex is thought to form an RNA ‘scaffold’ linked to reverse transcriptase, nucleocapsid, and possibly other viral and cellular proteins that together form the reverse transcription initiation complex [55,56]. A region called primer activation signal (PAS), located approximately 50 nucleotides upstream of PBS, enhances the utilization of tRNA_3_^Lys^ and appears to regulate the initiation reaction [57,58].

A part of the complex formed by HIV OT is viral integrase. It is known to be bound in a single complex with CA and RT and, apparently, is required for full-fledged reverse transcription. However, interactions affecting IN activity will be discussed in the next section.

It has also been shown that DNA topoisomerase 1 (TOP1) directly interacts with HIV-1 NC, which itself is required for the initiation of reverse transcription [59,60]. In addition, in vitro reverse transcription studies have demonstrated that TOP1 can significantly enhance HIV-1 OT activity, and this can be prevented by the addition of the TOP1 inhibitor camptothecin [59]. It is known that TOP1 can dissociate OT from structured RNA in an ATP-dependent manner, but the mechanism of this interaction has not yet been identified [61,62,63].

In addition to RTIC, a number of other cellular factors regulate the reverse transcription process. APOBEC3G (A3G) and A3F are known to inhibit the reverse transcription process [64,65]. Cellular expression of A3G leads to its incorporation into vif-deficient HIV-1 particles, whereas its presence in wild-type virions is dramatically reduced due to Vif-induced degradation via the ubiquitination-proteosome pathway [66]. The process of inhibition of OT by the A3G protein is not fully understood. There are several models of how this occurs, and these models may coexist with each other. First, it has been suggested that A3G does not inhibit the synthesis of new DNA but leads to the degradation of existing DNA. Thus, since the small amount of negative strand cDNA that is produced in newly infected cells (~5% of wild-type) contains 1–2% of cytosines deaminated by A3G to form uracil, it has been suggested that most of the newly synthesized viral DNA edited in this way will be degraded by the DNA repair system [67]. However, it is known that this mechanism cannot fully describe the activity of A3G against HIV reverse transcription. Several publications have shown that mutant A3G and mutant A3F, which have lost their cytidine deaminase activity, still show strong activity against HIV-1 and reduced viral DNA synthesis [68,69].

Another model is based on the fact that A3G interacts directly with RTIC and blocks the synthesis of a new DNA strand. One report [65] showed that A3G did not affect the kinetics of NC-mediated annealing reactions and did not inhibit cleavage by RNase H. In stark contrast, A3G significantly inhibited all OT-catalyzed DNA elongation reactions with or without NC. Fluorescence anisotropy and stretching analyses of individual DNA molecules showed that NC has higher nucleic acid binding affinity than A3G but, more importantly, exhibits faster association/dissociation kinetics. RT binds to single-stranded DNA with much lower affinity than NC or A3G. These data support a novel mechanism of deaminase-independent inhibition of reverse transcription that is determined by critical differences in the nucleic acid binding properties of A3G, NC, and RT.

An interesting factor affecting RT is the host dNTP-triphosphohydrolase (dNTPase), known as the protein containing the SAM domain and HD domain (SAMHD1). This protein limits the availability of dNTP substrates for HIV cDNA synthesis. The resulting dNTP-poor environment resulting from SAMHD1 suppresses both RNA- and DNA-dependent synthesis of HIV-1 proviral DNA and reduces the fidelity of reverse transcription [70]. SAMHD1 dNTPase activity limits HIV-1 replication in non-dividing cells such as macrophages, resting CD4 T cells, and dendritic cells [70,71,72,73,74]. Notably, SAMHD1 is able to repress HIV-1 LTR-driven gene expression in dividing cells, a function that is absent in both phosphorylation (T592A) and catalytic site mutants. However, this effect is likely due to changes in nucleic acid binding capacity rather than dNTPase activity, as dNTP levels are already high in this cell type [75].

In addition to the aforementioned proteins, there are a number of cellular factors that have been shown to be associated with the reverse transcription process, but the exact mechanism of their influence has not been established. These proteins include human antigen R (HuR) and A kinase anchor protein 1 (AKAP1) [76].

### 3.5. Integration

The resulting cDNA is integrated into the host cell genome by the enzyme integrase (IN), which forms a preintegration complex (PIC) with cellular proteins. Integration is an important step in the HIV life cycle. Recent studies have shown that the host protein POLE3 is a repressor of unintegrated viral cDNA transcription. This protein binds to DNA and prevents transcription factors from binding to the promoter region, and inhibition of POLE3 interrupts the viral life cycle, confirming that efficient replication can only occur when successfully integrated copies of HIV cDNA are transcribed [77].

The integrated viral DNA is called provirus, which can be used for transcription or remain in a latent state until it is activated by the host cell. HIV-1 IN is one of three viral enzymes. It belongs to the superfamily of polynucleotidyltransferases [78]. Polynucleotidyltransferases catalyze the transfer of nucleic acids. In the case of IN, the transfer of viral cDNA into the host cell genome is catalyzed. To do this, integrase, in complex with other factors (cDNA, viral proteins, cellular proteins), as the ‘preintegration complex’ (PIC), catalyzes the cleavage of dinucleotides from both 3′-ends of cDNA (this process is termed 3′-processing), binds the cellular DNA target, and performs the chain transfer reaction. The latter is the insertion of each of the 3′-ends of the processed viral DNA into each strand of cellular DNA [79]. The composition of PIC is not fully understood, but it is known that HIV integrase is the leader among viral proteins in terms of the number of interactions with cellular proteins.

Acetylation and deacetylation of nonhistone proteins play an important role in a multitude of cellular processes because they can affect protein function. The possibility of non-enzymatic acetylation of proteins has also been hypothesized [80]. Direct interactions between histone acetyltransferase (HAT) and histone deacetylase (HDAC) protein families with HIV-1 IN have been found.

HAT p300 directly binds to HIV-1 integrase and acetylates it at the C-terminal domain residues K264, K266, and K273 [81,82]. Acetylation of p300 enhanced the DNA-binding activity of IN and the efficiency of the chain transfer reaction in vitro, without affecting 3′-processing. In one work [81], a reduction in replication for viral variants with K(264,266,273)R mutant IN was shown to be similar to that with p300-specific inhibitors. However, these results were left in doubt by work where an HIV-based vector encoding IN carrying the same triple mutation was replication-competent [82].

It should be noted that in the work indicating impaired integration [81], the HIV-1 Bru isolate contained a FLAG-tagged IN located at the end of the C-terminal domain of the IN immediately adjacent to the acetylation target site, whereas in the other work [82] IN was not tagged. This fact may explain the contradictory results of these works because although the labeled wild-type virus is viable, the combination of this FLAG-tagged IN with a K(264,266,273)R substitution can significantly reduce the integration efficiency. Nevertheless, some effect of K(264,266,273,273)R substitutions on IN activity clearly exists. For example, even in the case of unlabeled IN, the presence of this triple substitution resulted in a 1.7-fold decrease in the amount of integrated DNA and a 4-fold decrease in 2-LTR circles, signaling less efficient integration than for wild-type IN [82]. The negative effect of K(264,266,273)R substitutions on integration was also confirmed in a different work [83].

There are conflicting reports on the interaction of another HAT, general control non-depressible 5 (GCN5), with HIV-1 IN. A group of researchers showed that it acetylates it at K258, K264, K266, and K273 [84]. Acetylation promoted both 3′-processing and chain transfer reactions in vitro. Viral strains containing IN K(258,264,266,273)R and K(264,266,273)R mutants showed a five-fold reduction in infectivity compared to the wild type, which resulted from reduced integration. A comparative analysis of replication efficiency of triple and quadruple mutant IN viruses convincingly showed that acetylation of K264, K266 and K273 by both GCN5 and p300 is necessary for efficient viral integration, whereas specific modification of K258 does not affect the interaction with GCN5 [58].

A different paper [85] describes receiving exactly the opposite results. The role of K258, K264, K266, and K273 substitution was investigated using the pNL4.3R-E-luciferase reporter viral vector. Luciferase levels were significantly decreased by both K258R (3-fold reduction) and K(258,264,266,273)R mutations. However, single K264R, K266R or K273R mutations had no effect. Quantification of total reverse transcription products and 2-LTR circles unexpectedly demonstrated a 3-fold reduction in total reverse transcription products and comparable levels of 2-LTR circles for both K258R and quadruple mutants. This result allowed the authors to consider that all K to R substitutions had no significant effect on HIV integration but could not explain the 50-fold decrease in luciferase reporter gene expression in the case of the quadruple mutant.

Further studies showed that the observed effect of the K(258,264,266,273)R mutant could be explained by its effect on early transcription events after integration, as there was an average 80-fold reduction in the level of transcripts produced by proviruses integrated by the IN mutant versus wild-type IN. Thus, the not active chromatin state present on unintegrated viral DNA can remain intact for a longer time, causing low provirus transcription even after integration [85].

Data on the interactions of HIV-1 integrase with HDAC1 are also contradictory. A number of studies show the effect of HDAC1 overexpression on the efficiency of HIV DNA integration, while HDAC1 knockdown has no effect on this process [86,87]. However, another group achieved the opposite result, as HDAC1 knockdown in HeLa cells increased the amount of integrated DNA and decreased the number of 2-LTR circles in infected cells. Treatment of cells with a potent inhibitor of mammalian histone deacetylases I and II, Trichostatin A (TSA), or a specific inhibitor of HDAC1 catalytic activity, MS-275, also increased the level of integrated DNA [87]. Another study showed the necessity of virion-associated HDAC1 activity to preserve the infectivity of these virions in target cells [88].

The observed discrepancy in the results on the effect of HDAC1 on HIV-1 replication can be explained both by different experimental conditions in different studies and by the fact that, in the cell, HDAC1 interacts with a large number of proteins and is a member of various complexes, the components of which can also interact with IN. For example, HDAC1 is associated with the Sin3a complex, which also contains Sin3a-associated protein 18 (SAP18) [89], which has been shown to interact with IN [88]. Given that HIV-1 IN binds SAP18, and selectively recruits components of the Sin3a-HDAC1 complex into HIV-1 virions, it is difficult to clearly understand which factor influences the infectivity of these virions in target cells.

Unlike HDAC1, the effect of HDAC10 on HIV replication is not related to deacetylation of IN. HDAC10 levels are reduced during HIV-1 infection due to the activity of the envelope glycoprotein associated with the virus [90]. Knockdown of HDAC10 in CD4^+^ T cells by specific shRNAs has been shown to enhance HIV-1 infection, in particular by facilitating the integration stage [91]. HDAC10 was found to interact with IN, and its binding site is located in the region of residues 55 to 165 of the IN catalytic domain. Importantly, HDAC10 did not significantly alter the lysine acetylation state of IN but weakened its interaction with LEDGF/p75. Since this interaction is important for IN stability and efficient integration into actively transcribed regions of the genome, its disruption led to impaired viral integration, which explains the negative effect of HDAC10.

Another partner of IN involved in the regulation of its deacetylation, together with HDAC1, is the cellular protein tripartite motif-containing 28 (TRIM28) [87]. TRIM28 is an epigenetic corepressor that is recruited to its targets by Krüppel-associated box domain zinc finger proteins (KRAB-ZFPs). TRIM28 then mediates transcription repression by recruiting histone modifying repressive factors, such as SET domain bifurcated 1 (SETDB1) methyltransferase, nucleosome remodeling deacetylase (NuRD) complex, and heterochromatin protein 1 (HP1). Together, they repress mobile elements [92]. TRIM28 is known to suppress HIV-1 gene expression and is involved in the establishment and maintenance of the latent state of the virus [93,94].

Protein phosphorylation is a reversible post-translational modification of proteins carried out by protein kinases that modify amino acids by adding a covalently bound phosphate group. The amino acids most commonly phosphorylated in eukaryotes are serine, threonine, and tyrosine. These phosphorylations play important and well-studied roles in signaling pathways and metabolism. However, several other amino acids (His, Arg, Lys, Asp, Glu, Cys) can also be phosphorylated [95]. Phosphorylation rapidly and reversibly regulates cellular signaling functions, affecting catalytic activity, protein:protein/DNA/RNA interactions, protein localization, and stability [96]. To date, two protein kinases, c-Jun N-terminal kinase (JNK) and general control non-derepressible-2 (GCN2), have been shown to phosphorylate HIV-1 IN [97].

Manganaro and colleagues found JNK-mediated phosphorylation of IN when studying the causes of latent HIV infection in resting peripheral blood lymphocytes (PBLs) [97]. They observed that several mitogen-activated protein kinases (MAPKs) are neither expressed nor active in resting CD4^+^ T cells but are activated in response to T cell stimulation. Inhibition of one of them, JNK, significantly impaired viral infection in phytohemagglutinin-interleukin-2 (PHA-IL2)-activated T cells. Inhibition of JNK resulted in increased early and late reverse transcripts but decreased HIV-1 integrated DNA and circular 2-LTR during infection with activated PBLs. These observations suggest that JNK activity is required for efficient integration [97].

JNK has been shown to interact with integrase in vitro and in vivo and to phosphorylate it via the highly conserved Ser57 located in the core domain of IN [97]. The phosphorylated IN becomes a substrate for a cellular peptidyl-prolyl cis/trans isomerase (PPIase) enzyme, Pin1, which catalyzes the conformational modification of integrase [98]. Pin1 is the only known PPIase that specifically binds phosphorylated S/TP motifs and catalyzes the isomerization of the peptidyl-prolyl bond. Isomerization can affect the folding, localization, activity, stability, etc., of the target protein [98]. In the case of INs, such isomerization also occurs. This significantly affects the stability of INs, as the S57A mutant was less stable than wild-type INs when overexpressed in HeLa cells. Inhibition of Pin1 resulted in reduced stability of wild-type IN but not the S57A mutant. This indicates the importance of S57 phosphorylation in maintaining IN stability and the role of Pin1 in this process [97].

In addition to JNK, IN has been shown to be phosphorylated in vitro and in vivo by GCN2 [99]. GCN2 is a serine/threonine kinase that is responsible for recognizing amino acid deficiency and triggering an associated integrated stress response. Upon sensing amino acid starvation, GCN2 changes its conformation, allowing autophosphorylation. The autophosphorylated form of GCN2 is its active form, which is able to induce a further integrated stress response [100].

GCN2 has been shown to phosphorylate IN at S24 and S255, and the latter has been identified as a major phosphorylation site in vitro [99]. Serine-alanine and serine-aspartic acid mutations at positions 24 and 255 did not alter the level of total DNA, IN stability or localization, nor did they alter selectivity for certain genomic features or histone modifications upon integration. In contrast, viral vectors carrying IN S(24,255)A and S255A mutants showed increased infectivity and integrated DNA levels upon single infection in HEK293T cells, whereas the IN S24A substitution had no effect.

PIC is localized in the nuclear periphery, which is a complex and heterogeneous structure. HIV-1 is known to integrate its genetic material into fragments of the cellular genome undergoing active transcription [101]. Analysis of approximately 1 million integration sites in HEK293T cells transduced with a single-cycle HIV-1 vector showed that almost 75% of them are located in active regions of the genome [102]. The fact that integrase itself is not specific for any particular sites in cellular DNA [79,103] suggests that there are cellular partners that, for example, are able to recognize certain epigenetic marks, thereby promoting integration into specific sites.

The choice of viral integration site is driven by numerous cellular factors. One of the most well-described partners of IN is lens epithelium-derived growth factor/p75 (LEDGF/p75). In the cell, it acts as a coactivator of transcription of stress-related proteins and protects the cell under various stress conditions [104,105,106]. LEDGF/p75 also plays a role in homologous DNA recombination [107]. The involvement of LEDGF/p75 in HIV-1 infection includes targeting the preintegration complex to actively transcribed regions, influencing IN localization, and influencing IN stabilization. LEDGF/p75 reduces polyubiquitination of IN, thereby increasing its stability. Interestingly, the stabilizing effect of LEDGF/p75 on IN was observed both in the nucleus and cytoplasm using wild-type LEDGF/p75 with nuclear localization and a mutant variant with cytosolic localization [108].

In 2007, IN positions W131, I161, R166, Q168, and E170 were found to be responsible for interaction with LEDGF/p75 in vitro [109]. It should be noted that mutations at these positions do not reduce the enzymatic activity of IN. In vivo experiments showed that in addition to E170, IN positions H171 and L172, located in the region of loop 170 of EHLK 173 between helices α4 and α5, were responsible for LEDGF/p75 binding [110]. These IN mutations significantly impaired its interaction with LEDGF/p75 but did not significantly affect the ability of IN to bind chromatin. Therefore, there may exist alternative mechanisms of IN binding to host chromatin.

In addition to influencing integration site selection, LEDGF/p75 may also contribute to stabilizing the functional multimeric state of IN. IN is known to function as a tetramer [111,112], and the dimer–dimer interface is important for tetramerization [113], coordinated in vitro integration, and HIV-1 infectivity. LEDGF/p75 is able to bind both the IN dimer and tetramer [114,115] and also promotes multimerization of wild-type IN and its mutant variants with reduced ability to multimerize: H12N, E11K, Y15A, D25A, K186E, K188D, and I191E [116].

Hepatoma-derived growth factor-related protein 2 (HDGFP2) also contains PWWP and IBD domains responsible for its interaction with trimethylated histone H3K36me3 and IN, respectively. While decreasing cellular levels of LEDGF/p75 reduced integration efficiency, HIV-1 replication remained at wild-type levels in cells with low HDGFP2 levels. When both HDGFP2 and LEDGF/p75 were depleted, the level of integration into active regions of transcription was lower compared to cells where LEDGF/p75 was depleted separately [117,118]. HDGFP2 appears to provide rudimentary integration in LEDGF/p75 knockdown cells, although its efficiency is lower than in control cells. Interestingly, overexpression of HDGFP2 in LEDGF/p75 knockdown cells restored HIV-1 replication and integration to wild-type levels [93,119].

Historically, integrase interactor 1 (INI1) was the first identified partner of IN. INI1 is a component of ATP-dependent chromatin remodeling complexes SWItch/Sucrose Non-Fermentable (SWI/SNF) [120]. Both positive [121,122,123] and negative [124] effects of this protein on HIV-1 replication have been reported. Although INI1 may influence HIV-1 integration site selection in vitro, it remains unclear whether it is involved in this process during infection. INI1 as part of the SWI/SNF complex is thought to promote directed integration into the genome. SWI/SNF is responsible for nucleosome remodeling, thereby facilitating the access of IN to cellular DNA [125].

The human Ras-related protein (hRAD) 51, from the cellular homologous recombination DNA repair system, directly interacts with HIV-1 integrase in vitro and inhibits its catalytic activity both in vitro and in the yeast cellular integration system [126]. It has also been demonstrated that the formation of the active nucleofilament hRAD51 is necessary for optimal inhibition through the dissociation mechanism of the IN–DNA complex. This inhibition can be enhanced in HIV-infected cells by chemical stimulation of the endogenous hRAD51 protein [127]. Although chemical activation of hRAD51 stimulated its ability to inhibit integration, its inhibition enhanced integration efficiency. This indicates that modulation of HIV-1 integration depends on hRAD51 recombinase activity. According to the results obtained, hRAD51 fulfils different functions in the regulation of HIV-1 integration, exhibiting early inhibitory and late stimulatory effects [128,129].

Integration of viral DNA into the cellular genome results in the formation of single-stranded 5-nucleotide breaks in cellular DNA that flank the integrated viral DNA, as well as unpaired CA dinucleotides at the 5′-ends of viral DNA. Further successful viral replication requires repair of these DNA lesions [130]. The mechanism of post-integration repair has been extensively studied and numerous data indicate that it involves proteins of the non-homologous end joining (NHEJ) system, which mediates the repair of double-stranded DNA breaks [131,132,133,134,135]. A key component of NHEJ is the DNA-dependent protein kinase complex (DNA-PKcs). A double-stranded DNA break is recognized by the Ku70/Ku80 dimer, which recruits the DNA-PKcs catalytic subunit to the site of the break. As a result, a heterotrimeric DNA-PKcs complex is formed, after which the repair mechanism is initiated [136].

Integration does not lead to double-strand breaks. To initiate post-integration repair, the Ku70 protein (a component of the NHEJ system) binds IN directly. After knockdown of Ku70 in infected cells, post-integration repair was significantly suppressed, but reverse transcription and integration remained at the same level [131]. Coprecipitation allowed us to identify the binding sites of IN and Ku70. Specifically, the α6-helix of IN (amino acids 200–220) binds the N-terminal domain of Ku70 (1–250) [137]. The study also demonstrated that amino acids E212 and L213 in the α6-helix of IN are crucial for the interaction between IN and Ku70. The Ku70 residues involved in IN binding are S69, I72, S73, and I76. In the same work, a model of the IN–Ku70 complex was proposed, and a drug inhibiting the formation of the complex was developed. The crucial role of IN–Ku70 binding for successful post-integration repair, and viral replication in general, is supported by the fact that both processes were impaired in the case of a pseudovirus carrying E212A and L213A substitutions in IN. These impair its ability to bind Ku70 but not its catalytic activity [131].

In some works, the cellular complex Fanconi anemia complementation group I and D2 protein (FANCI-D2) was identified as another functional partner of IN that binds to its C-terminal domain [138]. Notably, two proteins in this complex (FANCI, FANCD2) are able to interact with HIV-1 IN. Knockout of FANCI or FANCD2 in HEK293T cells using CRISPR-Cas9 reduced the expression of the reporter gene encoded by the HIV-1 single-cycle vector. However, protein overexpression in the corresponding knockout cells restored reporter expression. The observed effects were independent of the effect of FANCI or FANCD2 knockout on cellular DNA replication, as they were the same in non-dividing cells in which the cell cycle was stopped by aphidicolin.

Similarly, the group of S. Fu demonstrated the effect of FANCI-D2 complex components on efficient integration of viral cDNA and no effect on reverse transcription. Based on the cellular function of the FANCI-D2 complex, and the fact that knockout of DNA polymerase or Flap nuclease downstream of FANCI-D2, reduces the levels of integrated HIV-1 DNA, the author suggested that these proteins may be responsible for repairing DNA damage caused by viral DNA integration. However, the decrease in integrated DNA immediately after the initiation of integration (12 hpi), and the increase in non-integrated 2-LTR circles (24 hpi or later) in FANCI and FANCD2 knockout cells, may indicate an effect of these proteins (and/or downstream targets) directly in the integration process, rather than in post-integration DNA repair. To confirm one of the possible hypotheses, it would be necessary to measure the efficiency of post-integration DNA repair, for example, using the qPCR assay described [139].

Important aspects of protein–protein interactions for the realization of the viral life cycle are those that determine the stability of the enzyme and PIC. Degradation serves to control protein quality and homeostasis [140]. The RING-type E3 ubiquitin ligase TRIM33 has been shown to interact with IN and is an important determinant of its degradation. Interestingly, in TRIM33 knockdown cells, both early and late HIV reverse transcripts were also slightly increased. This is probably due to the effect of IN on reverse transcription mentioned above. In multiple BRU HIV-1 infection experiments, TRIM33 knockdown also increased IN levels and promoted infection, whereas TRIM33 overexpression in KD cells impaired infection. All these data suggest that TRIM33 is a major mediator of IN degradation and support the effect of IN concentration on successful HIV-1 replication [141].

Another E3 ubiquitin ligase involved in IN degradation is the cytokine receptor-like factor 2 (CRLF2) and Von Hippel-Lindau protein (VHL) complex. It consists of the substrate recognition subunit of VHL protein, the cullin-2 framework protein, the adaptor proteins elongin B and elongin C, and the E3 ubiquitin ligase Rbx1 [142]. VHL has been shown to bind IN in the presence of VHL binding protein 1 (VBP1) [143]. VBP1 itself was initially identified as an IN partner using a yeast two-hybrid assay and confirmed by co-immunoprecipitation analysis in a co-overexpression system [144]. When VBP1, VHL or Cul2 were suppressed in HeLa cells stably expressing IN-HA, there was less polyubiquitinated IN-HA and its degradation was slowed [143]. These observations suggest that the CRLF2-VHL complex acts as a VBP1-dependent E3 ubiquitin ligase for IN. This reduces IN levels and can therefore be considered as a negative factor for HIV-1 replication.

Unlike the E3 ubiquitin ligases mentioned above, the hRAD18 protein positively regulates IN stability, although it possesses a RING-finger domain. The interaction between IN and the repair factor hRAD18 was detected by co-immunoprecipitation assay. The hRAD18 fragment from amino acids 65 to 226 was shown to be sufficient for IN binding. The study also revealed the colocalization of IN and hRAD-18 in the nuclear structures of cotransfected cells. Importantly, increased cellular levels of hRAD18 were accompanied by increased levels of IN. This indicates that hRAD18 may stabilize IN in cells, but the mechanistic details of this effect are currently not entirely clear [145].

This protein is thought to be involved in post-replication DNA repair [146]. The interaction between IN and hRAD18 proteins has been shown by co-IP in co-expressing cell lysates. Moreover, colocalization of IN with hRad18 in nuclear structures was observed in a subpopulation of co-transfected cells. It was shown that when overexpressed together with IN, hRAD18 was upregulated compared to when IN alone was overexpressed. This was true for IN with N-terminal Phe (wild type), Arg, and Met. This implies that stabilization was independent of the N-terminal rule [145].

### 3.6. Transcription

Following DNA-provirus integration, transcription of the viral genome, mediated by RNA polymerase II (RNAPII), is required to complete the viral life cycle and to produce progeny virions [147,148]. Initial transcription of the HIV-1 genome is controlled by the 5′-LTR and is dependent on host cell transcription factors binding to an array of cis-regulatory DNA elements in the LTR promoter [149,150].

#### 3.6.1. Structure of the LTR Promoter

**Figure 3:**
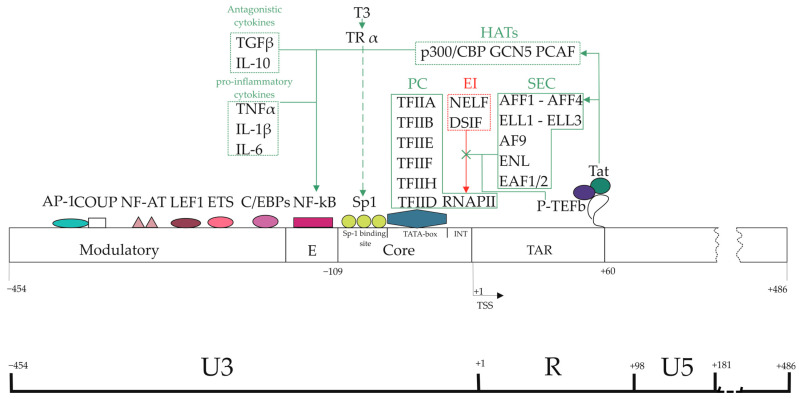
Structure and regulation of the LTR promoter. The HIV-1 LTR, indicating potential interaction sites for DNA- and RNA-binding proteins, is shown. Interactions between proteins are also shown. Green arrows mark interactions that enhance transcription. Red arrows mark interactions that inhibit transcription. Proteins circled with a solid line form a complex. Those circled with a dotted line act separately. E—enhancer, TAR—trans-activation response element, PC—preinitiation complex, EI—elongation inhibitors.

Transcriptional studies of the HIV-1 genome show that the 636 base pair HIV-1 LTR promoter can be divided into four functional domains (coordinates in relation to the transcription start site (TSS)): modulator element (−455, −104), enhancer region (−109, −79), core promoter element (−78, −1) and TAR region (+1, +60) (Figure 3).

The basal core promoter consists of a specialized initiator element, a canonical TATA element, and three tandem binding sites for specificity protein 1 (Sp1). The enhancer element contains two (one for some subtypes) neighboring binding sites for the inducible transcription activator nuclear factor kappa B (NF-κB). The modulator region contains elements for cell type-specific expression. The region after TSS, other than TAR, contains secondary enhancer elements and an unstably positioned nucleosome. Together, these elements act in concert to determine the level of HIV-1 transcription in a particular cell type.

#### 3.6.2. Involvement of Transcription Factors in Promoter Regulation

RNAPII-dependent transcription of protein-coding genes begins by recruiting gene-specific regulatory factors to the core promoter.

The TATAA element (located 25 bp upstream of the transcription initiation site) binds transcription factor II D (TFIID), which is a large multiprotein complex consisting of TATA-binding protein (TBP) and a number of TBP-associated factors (TAFs). TAFs function as adaptor proteins through protein–protein interactions and are required to mediate the response of transcriptional activators and repressor proteins [151]. TBP binding is the first step in the formation of the preinitiation complex and is often a critical rate-limiting step for RNAPII genes to be transcribed [152]. TBP binding to the LTR is necessary for the formation of the preinitiation complex and activation of viral transcription [153]. Upon TFIID binding, five more common TFs (TFIIA, TFIIB, TFIIE, TFIIF, TFIIH), the mediator complex and RNA polymerase II attach to the TATA box, together forming the preinitiation complex (PC) [154,155,156].

Immediately upstream of the TATAA element is a GC-rich promoter segment containing three binding sites for the ubiquitous transcription factor Sp1 [157,158]. Sp1, a zinc finger transcription factor involved in the transcription of many cellular genes, is crucial for both basal transcription and Tat-mediated trans activation of the viral LTR [159,160]. Sp1 binds its recognition site through three zinc finger motifs and activates transcription through a GC-rich hydrophobic activation domain [161]. Sp1 sites interact with the TATAA element, and increasing the distance between Sp1 sites and the TATAA element cancels HIV-1 replication [162,163]. Sp1 also cooperates with Tat during transactivation [164,165]. This interaction with Tat increases phosphorylation of Sp1 by DNA-dependent kinase [166]. These cooperative interactions indicate that the topological location of the three factors on the HIV-1 promoter is critical for HIV-1 expression. This indicates that Sp1, TFIID, and Tat must properly interact with each other for optimal viral transcription.

Sp1 binding sites have also been shown to interact with several other transcription factors. For example, BTEB, a GC-binding transcription factor, can bind to Sp1 motifs and activate the HIV-1 LTR [167]. Other members of the Sp1 family can also interact with Sp1 motifs but show differential effects [168]. Sp4 activates the LTR, and the Sp3 protein suppresses basal expression of the HIV-1 promoter. However, of the Sp1 family members, only Sp1 interacts with NF-κB to activate viral transcription, demonstrating a highly specific interaction between NF-κB and the trans-acting domain of Sp1 bound to a neighboring site. The GC-rich Sp1 binding sites incorporate a thyroid hormone response element that has been shown to mediate LTR activation by the thyroid alpha receptor [169].

Immediately upstream of the Sp1 binding sites are enhancer regions, which increase LTR transcription in response to various cellular activation signals [170]. Members of the NF-κB family (NF-κB1, RelA, c-Rel, NF-κB2, Re1B) bind as dimers to the HIV-1 enhancer, and certain combinations of NF-κB subunits are favored for Tat trans-activation [171]. Although binding of NF-κB to viral enhancer elements results in enhanced HIV-1 expression in T cells and macrophages [172,173,174,175], NF-κB sites are not important for HIV-1 replication [176,177,178,179] and appear to serve primarily to increase the rate of HIV-1 transcription during cell activation. The LTR enhancer is activated by pro-inflammatory cytokines including TNFα, IL-1β, and IL-6 [167,175,180,181,182,183,184,185,186]. Antagonistic cytokines such as TGF-β [187] and IL-10 can also stimulate the HIV-1 enhancer under certain conditions and require NF-κB binding activity [188,189].

The binding of TFIID and Sp1 is a key process for the initiation of transcription. Other elements are required mainly for RNA elongation and to increase the rate of transcription. Shortly after elongation, RNAPII stops and accumulates at the HIV-1 proximal promoter, producing only short transcripts. Due to the promoter proximal RNAPII pause, the HIV-1 provirus is ready for a rapid transcriptional response [190]. In this process, two strong inhibitors of negative elongation factor (NELF) and DRB sensitivity-inducing factor (DSIF) jointly stabilize the interaction of RNAPII with DNA, halting transcript elongation. In latent cells, the 3′-terminal processing protein Pcf11 is then recruited through direct interaction with NELF, which causes premature dissociation of RNAPII from the DNA matrix [190,191,192,193,194]. DSIF, a heterodimer of Spt4 and Spt5, is recruited to nascent RNA molecules and promotes capping [191,195,196]. Spt5 binds to newly formed RNA and in turn recruits NELF [197]. This creates a configuration in which the transcriptional complex has a limited chance of avoiding the proximal promoter, causing premature elongation termination in the range of a few hundred nucleotides [198]. Consistent with this, depletion of NELF leads to enhanced basal HIV-1 transcription and reduced latency [190,192,194].

In order for a productive viral replication cycle to occur, the transcription elongation block must be removed. For this purpose, the process of transactivation of transcription takes place. The LTR also encodes an RNA element, the trans-activation response element (TAR), located immediately downstream of the transcription initiation site. The TAR encodes a stem-loop structure of 59 nucleotides of RNA that forms a target at the extreme 5′-end of all HIV-1 transcripts for the virus-encoded trans-activator protein Tat [199,200]. Tat is an important viral regulatory protein that is expressed early in infection from multiply spliced viral mRNAs. By binding to TAR, Tat significantly increases the number of full-length transcripts [201]. Tat appears to be crucial for the rapid increase in genome transcription length required for the transition from quiescent to active viral infection. Expression of the viral Tat protein at a sufficient level disrupts NELF- and DSIF-dependent inhibition and induces the recruitment of coactivating factors, the most important of which are P-TEFb and SEC. In the TAR region, Tat and P-TEFb form a protein–RNA complex with the TAR RNA loop located at the 5′-end of all nascent viral transcripts. P-TEFb phosphorylates NELF [202], releasing it from the complex, and DSIF [203], converting it into a positive factor, counteracting the inhibitory activity of the complex on RNA polymerase II elongation [204,205]. SEC consists of scaffolding proteins (AFF1—AFF4) [206], elongation factors (ELL1—ELL3), and several additional partners (AF9, ENL, EAF1/2). AFF4 and AFF1 have short hydrophobic domains distributed along a flexible axis that mediates interactions with other subunits of the complex [207,208]. AFF4 directly contacts Tat and the surface of cyclin T1 [209,210]. The cooperative interaction between these subunits, AFF1 and AFF2 (which are in separate complexes), enhances Tat affinity for P-TEFb, with AFF1-SEC mainly involved in supporting Tat-mediated transactivation of the HIV promoter [206].

In addition, Tat itself can also activate NF-κB, forming a positive feedback loop to further enhance and maintain viral transcription [211,212,213]. For this, Tat-dependent activation of transcription is accompanied by the recruitment of transcriptional coactivators and chromatin remodelers, which also contribute to a significant enhancement of transcription from the HIV-1 promoter [205]. Tat has been shown to bind and attract HAT p300/CBP, GCN5, and PCAF to the 5′-LTR of HIV-1 [214,215,216,217,218,219,220], which by acetylating histone tails promotes a more accessible chromatin environment for transcription [221]. In addition to histones, HATs can acetylate and regulate TF activity [222]. For example, in Tat-dependent recruitment, p300/CBP acetylates the p50 NF-κB subunit [223], thereby increasing its binding capacity and likely its ability to enhance HIV-1 transcription in the context of the p50–p65 heterodimer [224]. Tat also interacts with TBP and Sp1 [225,226,227], and such cooperation is essential for HIV-1 LTR activation [228].

Transcription is also regulated in the modulatory region, predominantly through the recruitment of repressive factors. For example, several upstream binding sites for CCAAT-enhancer-binding proteins (C/EBPs) and activating protein-1 (AP-1) have been shown to mediate HIV-1 expression in some cell types but not in others [229,230,231,232]. Additional binding sites for various factors in this upstream region may also contribute to the regulation of HIV-1 transcription in lymphocytes, including E26 transformation-specific (ETS); lymphoid enhancer-binding factor 1 (LEF1); chicken ovalbumin upstream promoter (COUP); and nuclear factor of activated T-cells (NF-AT) [150].

In vivo and in vitro studies have shown that host cell transcription factors actively interact with the 5′-UTR [233,234,235]. The downstream binding sites of nuclear factor-1 (NF-1) and lipopolysaccharide binding protein (LBP) were originally identified by Jones and colleagues [236], but more than a dozen different regulatory elements within the 5′-UTR (that bind several different families of transcription factors) have now been identified [234,237,238]. These sequence elements include binding sites for both constitutive (i.e., Sp1) and inducible transcription factors (i.e., AP-1, NF-κB, NF-AT). Some of these have been shown to transmit activation signals to the LTR [239,240,241,242], and some are required for efficient HIV-1 expression and replication in T cells [238]. In particular, several AP-1 binding sites, which can also bind the ‘activating transcription factor/cAMP response element binding protein’ (ATF/CREB), have been shown to mediate cellular activation signals transmitted through the cAMP activated protein kinase (cAMPK) pathway [239,241]. The 5′-UTR also interacts with the unstably located nucleosome (Nuc-1), which is displaced during chromatin remodeling of the HIV-1 promoter [243].

Taken together, these studies suggest that responsive elements in the 5′-UTR may contain a downstream enhancer domain that can act independently, or in concert with the upstream enhancer and promoter, to maximize activation of HIV-1 gene expression. Acquisition of the downstream enhancer can expand the viral response to cellular activation signals and activate LTR transcription in response to a wide range of cellular activation signals. The downstream enhancer may also promote displacement of the unstable nucleosome and remodeling of the proviral chromatin structure.

Some studies show that transcription factors of the basic leucine zip (bZip) family play an important role in the regulation of HIV-1 transcription [231,244]. The bZip transcription factors include the AP-1 (Jun, Fos), CREB/ATF, and C/EBP families. These factors are nuclear phosphoproteins that combine to form homo- or heterodimers via their leucine zip domains [245]. The downstream region of the HIV-1 LTR contains three functional AP-1 binding sites that are important for viral transcription and replication [234,243,246]. These AP-1 sites bind purified c-Jun in vitro [234,246], demonstrating that these elements are true binding sites.

Interestingly, supershift analysis using nuclear protein extracts shows that AP-1 complexes consist of cFos and JunD as well as CREB, ATF-1, and ATF-2 [241,242]. This suggests that CREB/ATF proteins bind to AP-1 sites in cells as interfamily heterodimers with AP-1 subunits. Some AP-1 and CREB/ATF family proteins have been shown to undergo cross-dimerization in vitro [247]. For example, cJun and ATF-2 dimerize by binding to a mismatched AP-1 binding site in the promoter of the c-jun gene [247,248]. Consistent with cross-dimerization between AP-1 and CREB/ATF subunits, AP-1 sites were found to mediate cooperativity between TNFα and cholera toxin, a potent activator of the cAMPK pathway [239].

The LTR also contains several C/EBP binding sites upstream of the enhancer [249,250]. C/EBP sites in the HIV-1 LTR are critical for HIV-1 expression in macrophages, but not in T cells [230,231,232], whereas C/EBP inhibits HIV-1 expression in brain cells [251]. The C/EBP binding site can also interact with the neighboring NF-κB binding site to enhance HIV-1 gene expression [252]. This transcriptional synergy may be mediated by the cooperative binding of factors to the HIV-1 promoter, insofar as C/EBP has been shown to physically interact with transcription factors of the NF-κB family [253].

### 3.7. Post-Transcriptional Changes in mRNA

HIV-1 mRNA biogenesis requires the host cell RNA splicing machinery to produce fully spliced mRNAs that are transported from the nucleus to the cytoplasm by the endogenous cellular pathway. The HIV-1 primary transcript undergoes extensive alternative splicing that preserves the equilibrium of more than 50 mRNA isoforms, allowing balanced expression of all viral proteins. A full explanation of this process and its regulation is not within the scope of this overview, but current reviews devoted to this topic can provide context [254,255,256]. Nevertheless, we will discuss host factors responsible for the regulation of this process in this section.

RNA is spliced while pre-mRNA is bound to a large complex of cellular factors called spliceosomes [257,258]. The efficiency of early splice complex formation is determined by the intrinsic strength of the 3′ splice site (ss) (splice acceptor, A) and subsequent 5′ ss (splice donor, D); it is further regulated by a number of cis-acting elements by their interaction with splicing regulatory elements (SREs). HIV-1 splicing controls include exonic splicing enhancers (ESE) and intronic splicing enhancers (ISE), which facilitate splice site recognition and selectively bind members of the SR (Ser-Arg) family of proteins. In addition, there are intronic and exonic splicing silencers (ISS, ESS) that inhibit splicing and are usually bound by specific members of the heterogeneous nuclear ribonucleoproteins (hnRNP) family.

Unspliced and incompletely spliced cellular gene transcripts are normally degraded in the nucleus. Recent studies have also shown that MDA5 specifically interacts with HIV-1 mRNA and that recognition of HIV-1 mRNA by MDA5 triggers activation of innate immunity in macrophages [259]. To circumvent these surveillance mechanisms, HIV-1 and many other retroviruses, including human T-cell leukemia viruses, express regulatory factors that facilitate the transport of intron-containing viral RNA out of the nucleus. The first of these factors to be discovered was the Rev protein of HIV-1, which interacts with a highly structured RNA element in the env gene called the Rev response element (RRE) [260,261]. Some other retroviruses, as originally shown for the Mason-Pfeiser monkey virus, do not contain a protein factor and simply encode cis-elements called constitutive transport elements that interact directly with cellular RNA export factors [262].

Transport of HIV-1 mRNA from the nucleus, like most cellular proteins and RNAs, occurs through nuclear pore complexes [263]. RRE-bound Rev interacts with the karyopherin family member chromosomal maintenance 1 (Crm1) via a leucine-rich nuclear export signal (NES) near the carboxyl end of Rev. Crm1, like other members of the karyopherin family, binds to cargo in the presence of a GTP-bound form of Ran GTPase. Upon export to the cytoplasm via the NPC, the bound GTP is hydrolyzed to GDP by the proteins Ran GTPase activating protein (RanGAP) and RanBP1. This destabilizes the Rev complex and releases factors from the RRE [264]. Rev then re-enters the nucleus by binding to the nuclear import factor importin-β [265].

Three-prime processing and polyadenylation of pre-mRNAs in multicellular animals involves recognition of upstream AAAUAAA and downstream GU-rich motifs surrounding the cleavage and poly(A) addition site. The AAAUAAA signal is recognized by cleavage and polyadenylation specificity factor (CPSF), and the GU-rich motif is recognized by cleavage stimulatory factor or cleavage stimulation factor (CstF). In addition, the mammalian cleavage factors CF1m, CF2m, and poly(A)-polymerase are required for the cleavage reaction [266,267].

Like most retroviruses, HIV-1 contains a duplicated set of core elements rich in AAUAAA and GU at the ends of repeat region found in both the 5′- and 3′-LTR. HIV-1 uses several regulatory elements to direct processing to the 3′-LTR cleavage site. First, the HIV-1 U3 sequence, which is upstream of the 3′-processing signal but not associated with the 5′-processing signal, contains upstream enhancer elements (USEs) that promote CPSF binding and enhance polyadenylation at the 3′-ends of HIV-1 transcripts [268]. Second, the poly-A 5′ and 3′ LTR processing sites are embedded in a region of secondary structure called the poly-A hairpin, located in exon 1 immediately downstream of the TAR hairpin structure. Factors that bind to sequences upstream of the AAAAAA site are thought to open the poly-A hairpin and allow preferential use of the poly-A 3′-LTR processing site [269]. Finally, the snRNP splicing factor U1 inhibits the 3′-processing and poly(A) site in the 5′-LTR by binding to the neighboring 5′ ss D1. Mutations of the 5′ ss D1, which attenuate U1 mRNA binding, allow the normally silent poly(A) site of the 5′ LTR to be utilized [270,271].

### 3.8. Translation of Viral Proteins

Translation initiation of eukaryotic mRNAs involves scanning from the 5′-cap until the AUG initiator is recognized in the appropriate Kozak consensus sequence. Because HIV-1 exon 1 contains many highly structured regions, including the TAR sequence, primer binding site, poly(A) hairpin, and RNA packaging sequences, the typical ribosomal scanning mechanism for translation initiation is eliminated. Moreover, some of the HIV-1 mRNA UTRs contain AUG sequences upstream of the authentic initiator AUG, which may interfere with translation initiation at the authentic AUG. All HIV-1 env mRNA species are bicistronic and have a vpu open reading frame upstream of the transcriptional pathway that overlaps the open reading frame of the downstream env.

Several mechanisms have been proposed for how these obstacles are circumvented [272]. HIV-1 may include an internal ribosome entry site (IRES), similar to those found in picornaviruses, which allows recognition of the gag initiation codon. In addition, HIV-1 and other retroviruses contain post-transcriptional control elements (PCEs) that bind to cellular RNA binding proteins and can act as enhancers to facilitate translation initiation. Gag translation can be enhanced by RHA, the DEIH helicase [273], and the RNA-binding proteins SRp40 and SRp55 [274]. An additional mechanism that is used to bypass the vpu open reading frame and allow efficient translation in the downstream env AUG involves 5′-cap-dependent ribosomal bypass, whereby the scanning ribosome jumps over large regions of mRNA before recognizing the correct initiation codon [275].

### 3.9. Virion Assembly and Egress from the Cell

Next, the translated proteins and viral RNA are transported to the cell membrane and assembled into capsid particles, followed by formation of the lipid envelope and detachment of the viral particle from the infected cell [276,277]. The mechanism of transport of Gag and other viral proteins to the cell membrane has not yet been fully elucidated. There are hypotheses about both passive transport of proteins through the cytosol and active transport of proteins to the membrane [278,279,280,281]. Nevertheless, the capsid assembly process, in which viral RNA, capsid and matrix proteins play a crucial role, has been studied in sufficient detail. An important step after capsid assembly is the dressing of the virus with the lipid envelope and the detachment of the already prepared particles from the cell. It is known that host cell proteins are recruited to realize these steps.

The targeting of Gag to the plasma membrane, and subsequent virion assembly, is known to be facilitated by the lymphocyte kinase (Lck) protein [282]. It is possible that Lck enhances HIV-1 assembly through phosphorylation of a complex of adaptor proteins. Our data indicate that the Lck kinase domain is dispensable for its effect on Gag HIV-1 assembly. Interestingly, Yousefi et al. reported an inverse relationship between Lck kinase activity and HIV-1 replication in T cell lines [283]. Thus, it is possible that Lck adaptor activity is mediated by direct binding rather than protein phosphorylation. Moreover, neither SH2 nor SH3 domains are required for the ability of Lck to promote the release of HIV-1 virus-like particles.

In addition, Lck indirectly interacts with several components of the cellular protein sorting pathway, including adaptor protein complexes (APc) type 1 and APc-3 [284,285]; the trans-Golgi network 38 and 41 isoforms [286,287]; Rab6 [224]; and atypical protein kinases [288]. Thus, Lck may regulate HIV-1 assembly by acting as an adaptor protein for these or other cellular and viral proteins, such as HIV-1 Gag. In addition, various ‘endosomal sorting complexes required for transport’ (ESCRT) proteins have been reported to be involved in viral assembly and release [289]. It is possible that Lck interacts with components of this complex to influence HIV-1 assembly at the plasma membrane. Alternatively, Lck may influence the kinetics of endocytosis of newly formed viral particles at the plasma membrane [290], as occurs with CD4. This possibility was not directly tested in the aforementioned study and requires further investigation.

At the C-terminus of Gag is p6, a 52-amino-acid protein that is best known for having late or “L” domains and providing packaging determinants for the Vpr protein. The first indication that p6 plays a role in particle detachment or release was the finding that deletion of p6 results in a defect in particle release, with particles remaining attached to the host plasma membrane (PM) by a thin stalk [291]. The critical domain regulating release was mapped to a small PTAP motif via site-directed mutagenesis [292]. Gag proteins of other retroviruses have also been found to have short motifs that promote viral release, including PTAP, PPXY and YPXL motifs. The ability to participate in very late events (particle release) in the viral life cycle led to the term “late domains” for these motifs [293].

Subsequently, several groups have shown that PTAP binds to the cellular protein of tumor susceptibility gene 101 (TSG101), a component of the ESCRT-I complex, thus linking Gag to the ESCRT apparatus [294,295,296]. The ESCRT apparatus is a multi-subunit membrane remodeling complex that plays a key role in the biogenesis of intraluminal vesicles within the multivesicular body (MVB) [297,298]. A second L domain is also found in p6 of HIV-1: a YPXL motif that binds to ALG-2-interacting protein X (ALIX), which is a component of the ESCRT-III complex [299]. TSG-101 plays a dominant role in driving particle release, but ALIX recruitment has also been found to promote HIV-1 replication in T cells and macrophages [300]. Through L-domain interactions with ESCRT components, Gag co-opts the cellular membrane rupture machinery from its role in the complex of the late endosome with the MVB to aid the release of viral particles from the PM.

A subset of ESCRT components is required for the release of HIV-1 and other retroviruses, while other ESCRT components may play a supporting role. Specifically, ESCRT-III components, including CHMP2 and CHMP4, as well as the ATPase ‘vacuolar protein sorting-associated protein 4′ (VPS4), are recruited to the site of particle budding, where they mediate the final membrane rupture event that separates the thin “neck” of the budding virion from the cell. Remarkably, ESCRT-III components are organized into helical filaments on the inner membrane of the budding particle neck, performing an ‘inside-out cut’ [301,302]. The recruitment of components of the ESCRT machinery to stimulate the release of HIV-1 particles has been summarized in numerous reviews [298,303,304].

Ubiquitination of cellular proteins serves as a signal for recognition by the ESCRT machinery, prompting the natural hypothesis that Gag ubiquitination may play a role late in the life cycle. Supporting this, fusion of ubiquitin to the Gag of equine infectious anemia virus (EIAV) may compensate for the lack of a late domain, and fusion of a deubiquitinating enzyme to HIV-1 Gag results in reduced particle release [305,306]. Although the role of Gag ubiquitination remains uncertain, these studies suggest a potential role in ESCRT recruitment.

Notably, HIV-1 Gag lacks a late PPXY-type domain that binds some Gag retroviral proteins to the ubiquitin ligase family known as ‘neural precursor cell expressed developmentally down-regulated protein 4′ (NEDD4). NEDD4L has been shown to stimulate HIV-1 budding and to eliminate late budding defects, indicating that K63-linked polyubiquitin chains play some role in particle release [307,308]. Mercenne et al. identified angiomotin as a host adaptor protein that binds to NEDD4L and associates it with Gag in developing particle buds [309]. Angiomotin depletion caused particle budding to stop at a stage prior to TSG101 depletion, resulting in the appearance of a crescent of buds on the membrane. This indicates that angiomotin serves as an adaptor of NEDD4L acting at a stage prior to ESCRT recruitment and function, and is additionally involved in ubiquitination in events leading to particle release. The structural basis for the interaction of PPXY angiomotin motifs with the WW domains of NEDD4L has been reported [310].

## 4. HIV Proteins and Various Immune Factors

HIV’s ability to evade the immune system is a major reason why it has persisted for decades [311,312]. HIV has various internal mechanisms designed to evade, or disengage, the host immune system [313]. Infection is characterized by progressive disruption of the immune system, leading to opportunistic infections, autoimmune diseases, and malignancies [314]. The virus affects CD4^+^ T cells, macrophages, and dendritic cells. This leads to their depletion and dysfunction, with CD4^+^ T-cell counts below 200 cells/mm^3^ considered a diagnostic sign of AIDS [314,315,316].

HIV can evade the host immune system by suppressing the expression of major histocompatibility complex (MHC) class I and II molecules, which are proteins required for antigen presentation and recognition by immune cells [317,318]. This occurs at the molecular level through several mechanisms, including the ability of HIV to interfere with the transcription and translation of MHC class I and II genes. This reduces the overall expression of these molecules on the surfaces of infected cells [319,320]. This effect is mainly mediated by the HIV-1 Nef regulatory protein [321], which is important for viral pathogenesis and therefore a potential target for antiretroviral drug discovery [322].

Nef interacts with the cytoplasmic tail of MHC I and II molecules and redirects them to the endocytic pathway for degradation [323,324]. One proposed mechanism for HIV-1 Nef-mediated suppression of MHC-I molecules on the cell surface is that Nef and the phosphofurin acidic cluster sorting protein 1 (PACS-1) protein combine to usurp the endocytic pathway of ADP ribosylation factor 6 (ARF6). This ostensibly occurs via a phosphatidylinositol 3-kinase (PI3K)-dependent process and suppresses cell surface MHC-I in the trans-Golgi network [325].

The HIV Vpu protein also promotes the degradation of MHC class I molecules. Vpu targets MHC I molecules for degradation by interacting with the host protein beta-TrCP, which recruits the E3 ubiquitin-ligase complex to tag MHC I for degradation in the proteasome. The Vpu protein [326,327] prevents the transport of newly synthesized MHC-I molecules to the cell surface where they are required for recognition by immune cells [328,329,330] by sequestering MHC-I intracellularly during the early stages of endocytosis and recycling [331]. Vpu prevents the transport of newly synthesized MHC I molecules from the endoplasmic reticulum to the cell surface by acting on the host protein tetherin [332].

Tetherin is a membrane protein that suppresses the release of viral particles from infected cells, thereby limiting viral spread. Vpu counteracts this antiviral mechanism by binding to tetherin and promoting its degradation via the proteasomal pathway [333]. By degrading tetherin, Vpu enhances the release of viral particles from infected cells [334,335]. However, some studies have shown that tetherin also plays a role in transporting MHC I molecules to the cell surface [336]. Tetherin interacts with newly synthesized MHC I molecules and promotes their transport to the cell surface where they can present viral antigens to the immune system [337]. Consequently, by directing tetherin degradation, Vpu disrupts the transport of newly synthesized MHC I molecules from the ER to the cell surface, resulting in reduced MHC I expression at the cell surface [338,339]. This impairs the presentation of viral antigens to cytotoxic T cells and thus helps the virus to evade immune surveillance [336]. As a result, the ability of the immune system to recognize and respond to HIV-infected cells is compromised, allowing the virus to evade immune clearance and persist in the host [328,329,330].

HIV can evade the host immune system by destroying virus-specific helper T cells, which are important for coordinating the immune response against the virus [313]. This occurs at the molecular level through several mechanisms [340]. First, HIV can directly kill infected helper T cells by inducing apoptosis or programmed cell death [341]. Second, HIV-infected cells can also induce killing of uninfected helper T cells through the release of viral proteins such as Tat, Nef, and gp120. These activate apoptosis pathways in nearby cells through several mechanisms such as activation of Fas, FasL, and TNFα expression [342], as well as reduction of Bcl-2 expression and activation of p53 [343]. Third, HIV proteins can induce cell death pathways by disrupting the normal function of cellular proteins and organelles such as mitochondria. This can lead to the death of infected and uninfected helper T cells [313,344,345]. Loss of helper T cells impairs the ability of the immune system to mount an effective response to HIV, allowing the virus to persist and replicate in the host [315].

Interferons are mainly produced and released by host cells such as immune cells (macrophages, dendritic cells, T cells) and non-immune cells (fibroblasts, epithelial cells) in response to viral infections, some bacterial infections, or other immune triggers [346]. Upon detection of viral particles, production of interferons by the host creates an antiviral response that suppresses viral replication through a mechanism of induction of antiviral protein expression and activation of immune cells [347,348,349]. However, the virus can dissociate this host defense mechanism [350] by blocking the ‘Janus kinase and signal transducer and activator of transcription’ protein (JAK/STAT) complex, resulting in the cessation of interferon I production [351,352].

The JAK/STAT pathway plays a crucial role in the regulation of inflammation during viral infections such as HIV [353]. Activators of this pathway can potentially help control HIV, but HIV Vpu and Nef disrupt JAK/STAT activation by stimulating IFN-α, reducing its induction [353]. HIV also promotes degradation of type I IFN JAK/STAT pathway components by suppressing the induction of specific interferon-stimulated genes (ISGs). Various viruses have mechanisms for evading JAK/STAT signaling, and abnormal JAK/STAT signaling is associated with dysregulation of the immune system [354].

## 5. Conclusions

It is evident that the HIV life cycle, and the development of associated infection, are directly involved with multiple human protein systems. Viral proteins actively recruit host cell resources to achieve the formation of new virions. The most complex and understudied is the HIV integrase preintegration complex, which of all viral proteins is the most actively involved in interactions with cellular systems. Nevertheless, there are gaps in the understanding of other protein–protein interactions between HIV and humans. Study of the late stages of the HIV life cycle, especially virion assembly and egress of new particles from the cell, remains relevant. Interactions between viral proteins and host immune elements, as well as the immune system’s suppression and/or compensatory adaptation under the challenging circumstances of infection, remain an important aspect within the study of infection development.

The abundance of host factors involved in the HIV life cycle is noteworthy. These mechanisms suggest the possibility of interrupting viral replication by breaking such interactions. To date, the development of drugs capable of inhibiting some of the aforementioned human cellular proteins (without harming the host) is a promising direction. The widest opportunities for such therapies relate to interactions within the processes of integration and transcription since they are acutely dependent on human cellular complexes.

However, many interaction mechanisms between HIV and the host cell remain undiscovered. In particular, despite the fact that the composition of the preintegration complex is well enough studied, there is insufficient information about the mechanisms of its formation and the role of individual components in the integration process. Furthermore, the processes of reverse transcription regulation in HIV are not fully understood. As such, we cannot draw definitive conclusions about the contribution of host factors to this process.

## Data Availability

Data are available on request from the authors.
